# Urolithiasis Problems in Finishing Pigs

**DOI:** 10.3390/vetsci10120688

**Published:** 2023-12-03

**Authors:** Joris Vrielinck, Geert P. J. Janssens, Ilias Chantziaras, An Cools, Dominiek Maes

**Affiliations:** 1 Faculty of Veterinary Medicine, Ghent University, 9000 Ghent, Belgium; geert.janssens@ugent.be (G.P.J.J.); ilias.chantziaras@ugent.be (I.C.);; 2 Veterinary Practice, Hospitaalstraat 38, 8906 Ieper-Elverdinge, Belgium

**Keywords:** calcium carbonate, struvite, urinary pH

## Abstract

**Simple Summary:**

The paper describes two cases of urolithiasis in finishing pigs on two farms (A and B). On both farms pigs died of urinary bladder rupture due to urethral obstruction with calcium carbonate calculi. An in-depth diagnostic examination to elucidate pathophysiological mechanismes consisted of analysis of mineral composition of feed, drinking water, mineral composition of urinary stones, blood parameters (minerals, a bone resorption marker, parathyroid hormone, vitamin D metabolites), biochemical urinalysis and microscopic examination of urinary sediment. Although mineral composition of feed and drinking water was similar on both farms urinary calcium and phosphorus excretion and composition of urinary crystals was different: low urinary phosphorus and high urinary calcium excretion and presence of calcium carbonate crystals in farm A, low urinary calcium and high urinary phosphorus excretion and presence of struvite crystals (magnesium ammonium phosphate) in farm B. Disturbances in calcium and phosphorus absorption and homeostasis was demonstrated but the examinations could not fully explain the pathogenesis. Further research has to focus on calcium and phosphorus levels in the feed, absorption and excretion of these minerals due to gut or urinary microbiota dysbiosis as well as on vitamin D content of the feed.

**Abstract:**

This paper describes cases of urolithiasis in fattening pigs on two farms (A and B). Bladder rupture due to urethral obstruction with calculi was the principal finding during the necropsy of the pigs. An in-depth diagnostic examination was performed to elucidate possible pathophysiological mechanisms, namely Fourier-transform infrared spectrophotometry (FT-IR) analysis of the uroliths, blood analysis (farm A: 5 samples, farm B: 10 samples) for assessing concentrations of minerals, the bone resorption marker cross-linked C-telopeptide of type 1 collagen (CTX), parathyroid hormone (PTH), and vitamin D components, biochemical urinalysis (farm A: 5 samples, farm B: 7 samples), microscopic examination of urinary sediment (Farms A and B: 7 samples each), mineral composition of the feed, and analysis of the drinking water. Calcium carbonate was the main component found in stones from both farms, and calcium carbonate and struvite were the main components found in crystals from farms A and B, respectively. On farm A, urinary calcium excretion and urinary pH were high; on farm B, urinary phosphorus was high and urinary calcium was low with a normal urinary pH. The mineral compositions of the feed and drinking water were similar on both farms and could therefore not explain the difference between the two farms. Disturbances in calcium and phosphorus absorption and homeostasis might have been involved in these problems. Further research should focus on the calcium, phosphorus, and vitamin D levels in the feed and take into account other factors, such as the absorption and excretion of minerals due to gut and urinary microbiota.

## 1. Introduction

In male finishing pigs, urinary calculi or uroliths in the urinary tract can lead to obstruction of the urethra, followed by bladder rupture and finally death [1,2]. Urinary calculi consist of minerals that can form crystals under specific pathological conditions. Pathological conditions are determined by drinking water supply, water consumption, and diet. Insufficient water consumption decreases urinary output and the solubility of urinary solutes and is an important risk factor for urolithiasis [3]. When a urinary mineral concentration exceeds its solubility, supersaturation, mineral precipitation, and crystal formation will occur, which is a driving force for calculi formation [4]. Dietary factors are important because diet influences urinary mineral composition and pH [5,6]. Other conditions that influence crystal formation are imbalances between promotors and inhibitors of crystal formation. Urinary organic and inorganic anions like citrate, pyrophosphates, and magnesium, and macromolecules such as osteopontin and Tamm–Horsfall proteins can delay stone-forming processes [7]. Urinary pH can act as an inhibitor and promotor in calcium urolithiasis. Acidic urinary pH is associated with calcium oxalate stones, and alkaline urinary pH is associated with the formation of struvite, calcium phosphates, and calcium carbonates [8,9]. The most important pathological conditions that can lead to struvite and carbonate urolithiasis are urinary tract infections caused by bacteria that can degrade urea into carbon dioxide and ammonia, which is further hydrolyzed into ammonium and bicarbonate. After binding with available ions, struvite or carbonate apathite is produced [10]. The well-known cystitis and pyelonephritis in reproductive sows caused by the urease-positive *Actinobaculum suis* bacterium is frequently associated with a huge amount of gritty bladder material microscopically defined as struvite [11,12].

This paper describes urolithiasis in finishing pigs on two different farms. The results of microscopical and biochemical blood and urine analyses on both farms differed considerably, suggesting a different underlying pathogenesis. The detection of urolithiasis at an early stage is difficult, so in practice, symptomatic preventive treatment consists of enhancing water consumption (more drinking nipples, lowering stocking density), optimizing the Ca/P ratio (1:1 to 2:1), and acidifying the urine with d,l-methionine or ammonium chloride when “alkaline” crystals and stones are formed [13]. Proper preventive dietary measures can only be implemented after elucidating the pathogenesis.

## 2. Materials and Methods

### 2.1. General Description of the Farms and Anamnesis

A general description of farms A and B with emphasis on the fattening pigs is shown in Table 1. Both farms, located in the western part of Belgium, were visited to collect proper farm information and for an in-depth examination. 

On both farms, the mortality rate ranged between 2 and 3%. Bladder rupture due to urethral obstruction with mineral concrements was the principal finding during the necropsy of the pigs. The problems occurred mainly in the last month of the fattening period in pigs at least 22 weeks of age. On farm A, the problems occurred during the first half of 2021, and on farm B, the problems occurred during the second half of 2020. On farm B, chalky urinary deposits were observed on the walls of the pens where female fattening pigs, originating from the same batch as the male pigs, were housed. 

Two different feeds were provided during the fattening period, one during the first half of the fattening period (for pigs until approximately 16 weeks of age) and a second feed for older fattening pigs (from 16 weeks onwards). 

Sodium chloride, as well as sodium bicarbonate, were supplemented to the feed of farm B, each at a dose of 2 kg/ton. This was performed as a preventive measure against stomach ulcers. 

On both farms, diets were supplemented with vitamin D, namely 50 µg/kg feed or 2000 IU/kg vitamin D. For pigs up to 60–70 kg, half of the vitamin D content was provided as vitamin D and half was provided as 25(OH)D. For pigs heavier than 70 kg, vitamin D supplementation consisted of 100% vitamin D.

### 2.2. Drinking Water Intake

On both farms, the drinking water intake (Lit/pig/day) could not be measured at the barn or compartment level. The overall consumption could be estimated by dividing the total daily water consumption (Lit/day) on the farm by the number of fattening pigs present on the farm. The daily drinking water intake per pig from a farm without urolithiasis problems in the fattening pigs was used as a control [14].

### 2.3. Drinking Water Quality

In each farm, one sample of drinking water was taken from the drinking nipple in the pen of the fattening pigs. The water was analyzed biochemically and microbiologically. The analyses were performed in the laboratory of Inagro, a research and advice center for agriculture in West Flanders, Belgium.

### 2.4. Feed Analysis

In each farm, one feed sample was collected during the urolithiasis problems, when the pigs were 20–24 weeks of age (feed of second phase). The samples were analyzed to determine the levels of macronutrients, macrominerals, and microminerals. Macronutrients were assessed using proximate analysis, and mineral composition was assessed using induction-coupled plasma spectrometry. The mineral composition of feed from a farm without urolithiasis problems was used as a control. 

The dietary base excess or cation–anion balance (CAB, mmol/kg DM) was calculated as the difference between the sum of the alkalizing ions (Na, K, Ca, Mg) and the sum of the acidifying ions (P, S, and Cl) using the following formula [15,16,17]:

CAB (mmol/kg DM) = 50 Ca + 83 Mg + 26 K + 44 Na − 59 P − 87 S − 28 Cl

The minerals are expressed as g/kg DM.

### 2.5. Blood Parameters

In the compartments where urolithiasis occurred, blood samples were taken by puncture of the jugular vein from 5 pigs on farm A and 10 pigs on farm B. After decanting the clotted blood, the serum was collected and stored at −20 °C until analysis. Different parameters related to calcium metabolism were analyzed. Cross-linked C-telopeptide of type 1 collagen (CTX), a bone resorption marker, and parathyroid hormone (PTH) were examined using the electrochemiluminescence immunoassay (ECLIA). The minerals Ca, P, K, and Na were analyzed using indirect potentiometry. Three vitamin D metabolites were examined using liquid chromatography–tandem mass spectrometry (LC–MS/MS), namely 25-hydroxycholecalciferol (25(OH)D, the metabolite used to determine vitamin D status, 1,25-dihydroxycholecalciferol (1–25(OH)_2_D, the biologically active metabolite, and 24,25-dihydroxycholecalciferol (24–25(OH)_2_D, an inactive vitamin D metabolite [18,19]. 

All analyses were performed in the Laboratory for Clinical Biology at the University Hospital of Ghent University, Belgium. Serum parameters of a control farm without urolithiasis problems were used as controls.

### 2.6. Examination of Bladders and Urine in Slaughtered Pigs from Farm B

At the slaughterhouse, 50 bladders of male finishing pigs from farm B were examined to assess the presence of macroscopic calculi. Urinary samples were macroscopically examined for the presence of stones and grit. The urine samples were not examined for the presence of microscopic crystals because a previous study showed that these crystals can be influenced by the slaughter process [14].

### 2.7. Analysis of Urine Samples Taken at the Farm

In both farms A and B, spontaneously voided urine samples were collected from pigs in the group with urolithiasis problems (7 pigs from each farm). Urine samples from farms A and B were taken in June and November, respectively. For this purpose, a 2-meter-long stick with a plastic drinking cup fixed at the end was constructed. 

The urine was evaluated macroscopically for color, clearness, and the presence of grit. After centrifugation of 2 mL of urine in a Statspin VT Rotor (Vetquip, Sydney, Australia) for 45 s at 9800 rotations per minute, the sediment was examined microscopically under two magnifications, 100× and 400×, for the presence of urinary crystals. The abundance of crystals was assessed at a magnification of 400× using the scoring system described by Vrielinck et al. [14], namely score 1 when 1 or 2 crystals were present per microscopic field, score 2 for 3–5 crystals, score 3 for 6–20 crystals, score 4 for 21–100 crystals, and score 5 for cases when the number of crystals was uncountable. 

The urine samples were analyzed biochemically for the determination of Ca, P, Mg, Na, Cl, and K by indirect potentiometry. Urinary citrate was determined using an enzymatic method, and urinary creatinine was photometrically determined. All values are expressed as standardized to creatinine (expressed as mmol/g creatinine). Analyses were performed at the Laboratory for Clinical Biology at the University Hospital of Ghent University, Belgium. Biochemical urinalysis data from a farm without urolithiasis problems were used as a control. 

### 2.8. Analysis of the Mineral Composition of Uroliths

Uroliths (stones) obtained during necropsies (farm A) and at the slaughterhouse (farm B) were analyzed and identified using semiquantitative Fourier-transform infrared spectrophotometry (FT-IR analysis). The FT-IR spectrum was compared with a standardized spectrum for biomaterials.

## 3. Results

### 3.1. Drinking Water Intake

The estimated drinking water consumption at the farm level was 5.5 and 6.5 Lit/pig/day on farms A and B, respectively. The average daily drinking water intake in fattening pigs of a control farm was 4.4 Lit/pig/day [14].

### 3.2. Drinking Water Analysis

The results of the biochemical analyses of the drinking water from farms A and B (Table 2) were similar for both farms, and values were below the maximum reference levels for the different parameters. The drinking water was bacteriologically contaminated with sulfite-reducing clostridia on farm A and with enterococci and sulfite-reducing clostridia on farm B.

### 3.3. Feed Analysis

The results of the feed analysis are shown in Table 3. No major differences in macro- and microminerals were observed between farms A and B and the control farm. The extended CAB was 376, 416, and 399 on farms A, B, and the control farm, respectively. 

### 3.4. Blood Parameters

CTX, Ca, K, and P concentrations were comparable between farms A and B (Table 4). CTX concentrations on farms A and B were lower than those in the control farm. Sodium concentrations were slightly higher in pigs from farm A than in those from farm B. The PTH concentrations were low on farm A, whereas the concentrations on farm B were similar to those in the control farm.

All mean 25(OH)D concentrations were within the reference range of 18–30 ng/mL for fattening pigs [18]. The 1,25(OH)_2_D concentrations were higher on farm A, and especially on farm B, than in the control farm. The concentrations of 24,25(OH)_2_D on farm B were lower than those on farm A and the control farm. All ratios of vitamin D metabolites were highest for farm B.

### 3.5. Urinalysis of Samples Taken at the Farm

All urine samples taken from farms A and B had a macroscopic turbid appearance before centrifugation for further microscopical analysis.

Most pigs on farm A had a high urinary pH (8.5). The specific gravity of the urine was similar to that of farm B. The main components of the urinary sediment crystals were calcium carbonate (calcite) and, to a lesser extent, calcium oxalate dihydrate. The urinary pH in pigs from farm B ranged between 7.0 and 8.0. Struvite (magnesium ammonium phosphate) was the most abundant crystal besides some calcite and amorphous material (Table 5). 

The urinary concentrations of Ca, Mg, K, and citrate in pigs from farm A were very high compared with those from farm B and the control farm (Table 6). On farm B, urinary P and Na concentrations were high, and citrate concentrations were low.

### 3.6. Mineral Composition of Uroliths

The urolith obtained during a necropsy on farm A consisted of calcium carbonate (100% calcite).

During the slaughterhouse visit to farm B, stones were found in 28 of 50 urinary bladders (56%) of male fattening pigs. Most of them were small (approximately 1 mm diameter) and varied from a few (1–10) to multiple (20–100) within a bladder. Larger stones (>0.5 mm diameter) were detected in a few bladders. Two bladders (1%) contained grit, and in 20 bladders (10%), no macroscopic uroliths were found. Infrared spectroscopy of a mixture of five stones demonstrated a mixed composition of 90% calcium carbonate and 10% calcium oxalate dihydrate.

## 4. Discussion

The urolithiasis problems in the older fattening pigs of the two farms were clinically relevant because they were responsible for an increased mortality rate. Calcium carbonate was the main component in crystals from the urinary sediment of farms A and B; the main components of the macroscopic uroliths found on farms A and B were calcium carbonate and struvite, respectively. Risk factor analysis for calcium carbonate urolithiasis in goats showed a seasonal difference, with more cases during summer, (more heat-induced water loss), fall, and winter (reduced water intake) in comparison with the spring. Because the fattening pigs were raised indoors, a seasonal influence on urolith formation is unlikely [9].

Sufficient water intake is crucial for the preventive management of urolithiasis [3]. However, reduced water intake was likely not a factor in the development of urinary crystals because the estimated water consumption on both farms was similar or even higher than the drinking water intake measured in a control farm, namely 2–2.25 L per kg daily feed intake [14]. This was also supported by the lower urinary creatinine levels on farms A and B (1.77 and 2.25 g/L) in comparison with the control farm (4.18 g/L). According to NRC recommendations [23], voluntary water intake in the case of *ad libitum* feeding is 2.5 L water per kg of feed, with 2 l/kg of feed as the minimum water requirement. Therefore, the daily water requirement was met in both farms. 

The biochemical parameters of the drinking water in both farms were within normal ranges. There was, however, some bacteriological contamination with sulfite-reducing clostridia (farm A) and enterococci and sulfite-reducing clostridia (farm B). The role of this contamination in the urolithiasis problems of the farms is unclear, but it was likely not important. Also, no gastrointestinal problems were observed.

### 4.1. Differences in Crystal and Stone Composition between the Farms

**Figure 1:**
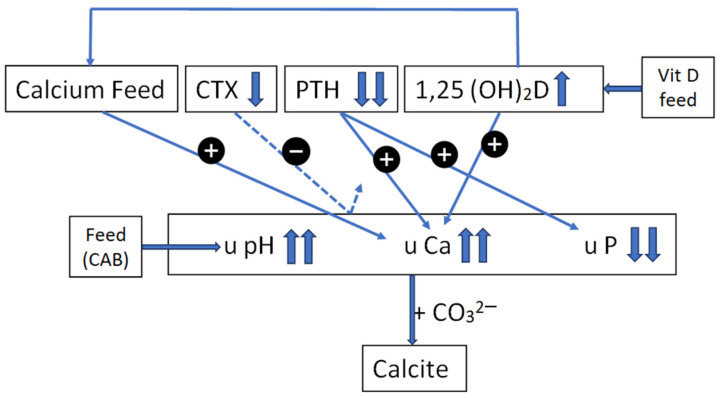
Farm A. Schematic representation of possible pathways and interactions between feed (calcium, vitamin D, cation–anion balance CAB) and blood serum parameters (CTX, PTH, 1,25 (OH)_2_D) on the urinary parameters (pH, Ca, P) and calcium carbonate crystal formation on farm A.

**Figure 2:**
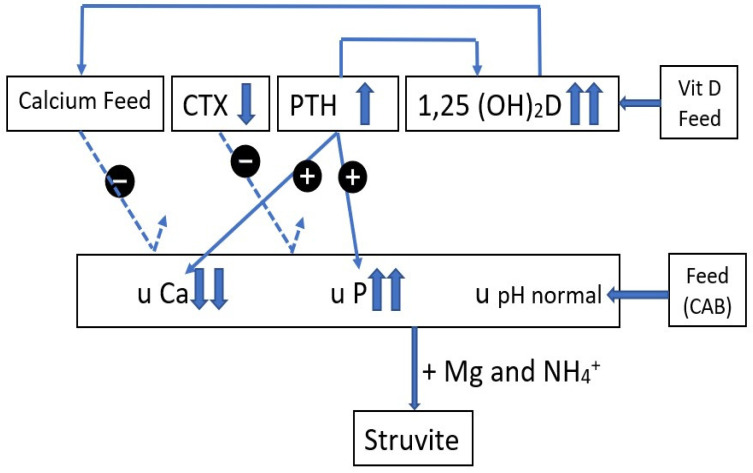
Farm B. Schematic representation of possible pathways and interactions between feed (calcium, vitamin D, cation–anion balance CAB) and blood parameters (CTX, PTH, 1,25 (OH)_2_D) on the urinary parameters (pH, Ca, P) and struvite formation on farm B.

The relationship between the different feed and blood parameters and urinary findings for farms A and B are schematically represented in Figure 1 and Figure 2. 

High 1,25 (OH)_2_D serum values can stimulate intestinal calcium absorption independent of PTH (low values) and cause absorptive hypercalciuria. The low serum values of CTX have no link with the high Ca and low P urinary values (dotted line). The high urinary pH cannot only be explained by the CAB of the feed. The low urinary P and high urinary Ca might be related to low and high absorption of P and Ca, respectively.

High 1,25(OH)_2_D values are partly controlled by PTH control and are not able to induce strong calcium intestinal absorption, leading to low calcium levels in the urine. The low CTX values have no link with the high P and low Ca urinary values (dotted line). The high P urinary excretion is controlled by PTH. Together with Mg and NH_4_^+^, struvite crystals can be formed. Low urinary calcium excretion is indicative of a disturbance in calcium intestinal absorption.

Calcite was the main stone component on both farms, but the crystals in the urine sediment were different: calcite on farm A versus struvite and, to a lesser extent, calcite on farm B. This discrepancy between macroscopic and microscopic findings is not unusual. Stones are a result of long-term exposure to supersaturated urine, whereas urinary crystals are related to the current urinary composition [24]. Also, the urinary mineral concentrations were different between the farms: for farm B, Ca, Mg, and K concentrations were lower than those of farm A, whereas farm B has the highest P and Na urinary concentrations. The mineral content of the feed was similar between both farms, except for Na and Cl, which were higher on farm B. The higher urinary concentration of Na on farm B was likely due to the supplementation of sodium chloride and sodium bicarbonate via the feed. In humans, dietary sodium chloride supplementation also leads to calciuria and increases the risk of calcium crystal formation [25,26] because calcium reabsorption parallels sodium reabsorption in the proximal tubules and loop of Henle. However, higher urinary sodium excretion on farm B did not lead to higher urinary Ca excretion. The higher dietary Na concentration on farm B might explain the lower urinary citrate concentration. In humans, salt loading is a twofold risk factor for urolithiasis, first by introducing calciuria (a condition that was not observed on farm B) and second, by the development of hypocitraturia, a risk factor for calcium oxalate urolithiasis. Citrate forms complexes with urinary calcium to form salts that are more soluble than calcium oxalates [27]. In adult humans, hypocitraturia is defined as a daily citrate excretion below 1.7 mmol [28,29]. Salt loading suppresses the renin–angiotensin–aldosterone system. Lower renin production suppresses final angiotensin II production, which reduces aldosterone production, which is involved in Na reabsorption in the distal tubule cells, and also suppresses the Na–H antiporter in the luminal membrane of the proximal tubule cell. Less Na will be reabsorbed and fewer protons excreted. As a consequence, the proximal intracellular proton concentration increases, and less citrate escapes into the urine, leading to hypocitraturia [30]. The differences between both farms relative to the sodium feed concentrations (farm A: 1.74 g/kg, farm B 3.83 g/kg) and urinary sodium concentrations (farm A: 26.85 mmol/g creatinine, farm B: 39.99 mmol/g creatinine) explain the difference in urinary citrate excretion (farm A 0.11 mmol/g creatinine, farm B: 2.29 mmol/g creatinine). In accordance with our previous research [14] and in contrast to findings in human studies [28,29], urinary citrate has no inhibiting influence on calcium crystal development. 

### 4.2. Differences in Urinary Potassium and Citrate Excretion between the Farms

Urinary concentrations of K and citrate were very high on farm A, although the K concentrations in feed and water were comparable between both farms. Likely, urinary pH was a determining factor. Citrate, a tricarboxylic acid, is the most abundant urinary organic anion with pKa values of 2.9, 4.3, and 5.6. So, at physiological blood pH and alkaline urinary pH, the trivalent citrate 3-anion is formed. Because citrate is reabsorbed in the proximal tubules only in the divalent ionic form (cotransport of 3Na^+^/citrate^2−^), citrate reabsorption will be lower at higher urine pH, and more citrate will be excreted via the urine. The opposite occurs in the case of acidosis [31]. In contrast to urolithiasis in humans, where urinary citrate is regarded as an inhibitor of crystal formation by forming soluble calcium citrate, the presence of citrate in the urine of swine is of no benefit in the prevention of calcite urolithiasis. The urine samples from farm A with calcite crystals had the highest citrate concentration. This is in accordance with the findings described in a previous paper [14].

### 4.3. Differences in Urinary Magnesium Excretion between the Farms

The higher urinary Mg excretion on farm A compared with farm B may be related to the different serum concentrations of PTH. The PTH hormone stimulates renal Mg conservation by increasing Mg absorption in both the thick ascending limb and the distal cortical duct of the kidneys [32]. Remarkably, no magnesium ammonium phosphate (struvite) crystals were detected in the urine samples from farm A. Low urinary phosphate excretion is probably the reason why phosphate calculi or struvite crystals are not formed. Struvite crystallization requires a high molar product of the three main components, namely phosphate, ammonium, and magnesium.

### 4.4. Differences in Urinary P Excretion between the Farms

The concentration of P in urine samples from farm A is low, whereas the opposite is seen in urine samples from farm B. The kidneys play a key role in phosphate homeostasis in order to maintain serum P concentrations within a narrow range [33]. As for Ca, this depends on the interaction between absorption in the gut, exchange with bone, and renal excretion. In the case of P restriction, phosphate resorption in the proximal tubule of the kidney is increased to conserve P homeostasis. On both farms, the concentrations of P were low, namely 3.81 g/kg on farm A and 3.77 g/kg on farm B. These low-P feeds are in accordance with the Belgian Feed Association convenant/agreement (covenant on low phosphorus and protein feeds and decreasing of phosphate and nitrogen in animal manure and environment) and are used to limit environmental pollution [34]. The Ca concentrations on farms A and B were 6.75 g/kg and 7.31 g/kg, resulting in Ca:P ratios of 1.77 and 1.93, respectively. These ratios are wider according to the recommended Ca:P ratio of 1.17 for growing pigs [23]. A Brazilian study on urolithiasis in pigs found much lower Ca:P ratios (0.35 and 1.05) together with low Ca concentrations (1.589 g/kg and 4.404 g/kg) [2]. In that study, calcium carbonate and magnesium ammonium phosphate were found, and the problems disappeared after the adjustment of the dietary Ca:P ratio to 2:1. On the farms in our study, Ca:P ratios were close to 2:1, and the causative factors for stone formation are different. Because P concentration in feeds is restricted, feeds are commonly supplemented with phytase to enhance the digestibility of organic P sources. Proper feed management procedures must be implemented for the phytase enzyme to be effective [35]. If this is not the case, P intestinal resorption might become critical, leading to relative hypophosphatemia. Indeed, P serum values for both farms were low, with the lowest values for farm A (2.67 mmol/L), suggesting disturbances in P homeostasis. If there is insufficient intestinal P absorption, the maintenance of phosphate and calcium homeostasis will be mediated by PTH and vitamin D. On farm A, PTH values were low, and because it is a phosphaturic hormone, P urinary excretion was low. The higher PTH serum values on farm B can explain the higher P urinary excretion.

### 4.5. Differences in Urinary Calcium Excretion on Both Farms

In contrast to farm A, where P homeostasis might have been disturbed, for farm B, reduced intestinal Ca absorption, possibly due to less digestible Ca sources, might have played a role in the problems. Concomitant relative hypocalcemia can trigger PTH production and, consequently, 1.25(OH)_2_D production by the kidney. This can explain the high phosphaturia and low calciuria on farm B. 

### 4.6. Calcium Metabolism on Farm A

On farm A, urinary calcium concentration was high and in line with the pronounced calcium carbonate crystal formation. The high urinary calcium excretion suggests disturbances in Ca metabolism. Ionized Ca and calcium-containing salts are filtered through the glomerulus and by transepithelial transport mechanisms and are normally reabsorbed by 98% [36]. Control of plasma Ca concentrations is under the control of PTH and vitamin D. PTH stimulates bone resorption, Ca gut resorption, and stimulates active Ca reabsorption in the distal nephron. Vitamin D, on the other hand, stimulates both Ca and P absorption from the gut and enhances bone resorption in concert with PTH. Absorptive hypercalciuria can possibly explain the calcite formation on farm A. In the case of absorptive hypercalciuria, there is high intestinal Ca absorption, resulting in enhanced filtered load and reduced tubular reabsorption in the kidney because PTH values are low. The values of CTX, a bone resorption marker, were low for both farms and even numerically lower than the CTX values in the control farm, which indicates that bone resorption was not involved in urinary stone formation in our cases and that urinary Ca on farm A originates from an exogenous (dietary) source. 

Enhanced renal synthesis of 1,25(OH)2D can contribute to absorptive hypercalciuria. Because the pigs are housed indoors, they are entirely dependent on the diet for vitamin D supply, which is regulated and restricted by the European Food Safety Authority to 50 µg/kg feed or 2000 IU/kg feed in total [37]. In cases of calcite urolithiasis, the supply of vitamin D might have to be lowered to limit excessive intestinal Ca absorption.

### 4.7. Factors Influencing the pH of the Urine

As for urinary citrate excretion, there is also a clear link between urolith and crystal formation and urinary pH. Phosphate has three pKa values: 7.2, 6.8, and 12.1. The pKa value for carbonate/bicarbonate is 10.3, so the phosphate in the struvite molecule is the HPO_4_^2−^ form because the Pka for PO_4_^3−^ (12.1) is out of the physiological range. The pKa of HPO_4_^2−^ is 6.8, so struvite crystal formation can start at a low pH. For calcium carbonate formation to start, much higher pH values are required (pKa 10.3.). Urinary pH is largely, but not only, determined by the CAB. Urinary pH can theoretically be calculated using the following equation: pH = 6.19 + 0.0031 CAB + 0.000003 CAB^2^ [15]. The calculated pH then becomes 7.77 for farm A and 7.96 for farm B, whereas urinary pH values of approximately 8.5 and 7.5 were observed on farms A and B, respectively. The differences might be due to other nutritional factors or the presence of bacteria in the urine. Bacteria can degrade urea into carbonate and ammonium, leading to an increased urinary pH. In a previous study, we never observed cystitis lesions in the bladders of finishing male pigs [21] Therefore, we did not perform a bacteriological examination of urine on the present farms. Research in humans has shown that even without visible signs of cystitis, the urinary bladder is not sterile and there is a urinary microbiome [38]. Although these microbiomes are difficult to detect using conventional urine culture protocols, they might influence the development of uroliths [39] by altering urinary pH to values different from those based on the cation–anion balance of the feed. Human research has shown that kidney stone patients have a characteristic gut microbiota dysbiosis that can affect stone formation [40,41]. The beneficial role of intestinal *Oxalobacter formigenes* in degrading intestinal oxalate and consequently reducing the renal oxalate burden is well-known [42]. 

On both farms, the problems disappeared after six months. No specific measures were undertaken on farm A, whereas farm B stopped the supplementation of sodium chloride and sodium bicarbonate. It cannot, however, be ruled out that during the six months after the study, changes in feed composition and digestibility of the nutrients occurred and led to a different urine composition that was less conducive for crystal and stone formation.

## 5. Conclusions

The urolithiasis problems of the two farms caused increased mortality in fattening pigs. Calcium carbonate was the main component in the urinary stones from farms A and B; the main component of the microscopic uroliths found on farms A and B were calcium carbonate and struvite, respectively. Traditional examination of feed and drinking water, blood parameters, and urine biochemistry could not fully explain the pathogenesis although disturbances in the calcium and phosphorus metabolism could be elucidated. Further research focusing on the calcium, phosphorus and vitamin D levels in feed and other factors such as absorption and excretion of minerals such as calcium and phosphorus due to gut or urinary microbiota dysbiosis is warranted to decrease and solve problems with urolithiasis.

## Figures and Tables

**Table 1:** General information of fattening pig farms A and B used in this study.

	Farm A	Farm B
Type of farm	Farrow-to-finish (20% of piglets are sold)	Fattening farm
Number of fattening pigs	4000	8000
Breed of fattening pigs	Topigs × Piétrain	PIC × Piétrain
Male pigs	Surgically castrated	Vaccinated against boar taint (Improvac^®^) at 12 and 20 weeks of age
Housing of males and females	Mixed in the same pen	In separate pens
Pen size (m^2^)	10	10
Stocking density (m^2^/pig)	0.85	0.72
Number of feeding places per pen	2	2
Number of drinking places per pen	2	1
Flow of drinking nipple	1 Lit /min	>0.5–1.0 Lit/min
Source of water	Deep pit (50 m)	80% phreatic water (3–5 m depth)/20% rainwater
Type of floor	Concrete, fully slatted	Concrete, fully slatted
Feeding	*ad libitum*	*ad libitum*
Type of feed	Mash	Pellets
Number of feeds during the fattening period	2	2
Mortality rate (%) during the fattening period	3	2–3

**Table 2:** Biochemical and microbiological analyses of drinking water from Farms A and B.

	Farm A	Farm B	Reference Values ^a^	
Biochemical Analysis			Minimum	Maximum
pH	8.1	7.8	4	9
Nitrate (mg/L)	7.3	18		200
Nitrite (mg/L)	<0.03	0.38		0.5
Bicarbonates (mg/L)	369	414		
Carbonates (mg/L)	0	0		
Chlorides (mg/L)	40	80		250
Sulfates (mg/L)	83	137		250
Total hardness (F°)	17.87	49.39		35.3
Ca (mg/L)	33	170		270
Mg (mg/L)	23.1	17		50
Na (mg/L)	116	46		400
K (mg/L)	10.8	4.4		
P (mg/L)	<0.05	<0.05		
B (mg/L)	0.34	<0.2		
Fe (mg/L)	<0.052	0.48		
Mn (mg/L)	<0.02	0.27		1
Cu (mg/L)	<0.02	<0.02		
Zn (mg/L)	<0.08	<0.08		
Microbiological analysis				
Bacterial count at 22 °C (CFU/mL) ^b^	39	1937		100,000
Bacterial count at 37 °C (CFU/mL)	8	297		100,000
Coliform bacteria (CFU/100 mL)	0	72		100
*Escherichia coli* (CFU/100 mL)	0	0		10,000
Enterococci (CFU/100 mL)	0	7		0
Sulfite-reducing clostridia	1	24		0

^a^ Reference values Animal Health Care Flanders, Belgium. ^b^ CFU: colony forming units.

**Table 3:** Results of the analyses of the feed (macronutrients and macro- and microminerals) for fattening pigs on farms A and B and a control feed [14]).

	Farm A	Farm B	Control Farm
**Macronutrients (%)**			
Dry matter	89.17	90.2	
Crude protein	16.11	15.77	
Ether extract	3.98	2.88	
Crude fiber	4.33	4.55	
Crude ash	4.74	4.80	
Nitrogen-free extract	60.01	62.2	
**Macrominerals (g/kg)**			
Ca	6.75	7.31	8.65
P	3.81	3.77	3.98
Na	1.74	3.83	2.21
Mg	2.11	1.52	1.76
K	7.68	7.64	6.36
Cl	3.08	3.75	4.04
S	0.64	0.83	0.56
**Microminerals**			
Cu, mg/kg	20	17	13
Fe, mg/kg	209	237	231
Mn, mg/kg	71	71	83
Zn, mg/kg	94	96	97
**Derived parameters**			
Na+K-Cl, meq/kg	185	256	145
Extended cation-anion balance; meq/kg	376	411	399
Ca:P ratio, g/g	1.77	1.93	2.17

**Table 4:** Blood serum concentrations (mean and median) of carboxy-terminal cross-linking telopeptide of type1 collagen (CTX), parathyroid hormone (PTH), minerals, and vitamin D metabolites in fattening pigs from Farms A and B and a control farm.

Parameter	Unit	Farm						
		A (n = 5)		B (n = 10)		Control (n = 30)		
		Mean	Median	Mean	Median	Mean	Median	Reference Interval
CTX	ng/mL	0.116	0.110	0.125	0.120	0.170	0.150	0.3 ^a^
PTH	ng/L	<1.20	1.20	4.20	3.1	5.43	2.87	
Ca	mmol/L	2.66	2.65	2.65	2.65	2.3	2.42	2.5–3.1 ^b^
K		5.56	5.55	5.58	5.51	6.57	6.17	4.8–7.8 ^b^
P		2.68	2.68	2.82	2.80	3.40	2.84	2.8–4.3 ^b^
Na		144.6	144.6	141.9	141.7	-	-	143–156 ^b^
25(OH)D	ng/mL	25.80	26.98	27.44	26.72	30.15	29.31	18–30 ^c^
1,25(OH)_2_D	pg/mL	95.27	91.36	110.49	112.75	78.54	74.22	
24,25(OH)_2_D	ng/mL	19.38	18.20	15.20	14.62	19.36	19.49	
25(OH)D:24,25(OH)_2_D	ng/ng	1.33	1.48	1.80	1.82	1.55	1.50	
1,25(OH)_2_D:25(OH)D	pg/ng	3.69	3.69	4.02	4.21	2.60	2.53	
1,25(OH)_2_D:24,25(OH)_2_D	pg/ng	4.91	5.01	7.26	7.71	4.05	3.80	

^a^ Geudeke et al., 2019 [20]; ^b^ Loynachan, 2012 [21]; ^c^ Madson et al. 2012 [22].

**Table 5:** Urinary pH, specific gravity (SG) of urine, and microscopic findings ^a^ of urine sediment in 14 urine samples (S) (7 from each farm) collected on-farm from fattening pigs on farms A and B.

.	Farm A				Farm B		
S	pH	SG (g/mL)	Microscopic Findings of Sediment (Score ^b^)	S	pH	SG (g/mL)	Microscopic Findings of Sediment (Score ^b^)
1	8.5	1.020	Calcite (5), COD (2)	1	7.5	1.015	Struvite (5), calcite (2)
2	8.5	1.021	Calcite (5),	2	7.5	1.016	Struvite (5), calcite (5)
3	8.5	1.022	Calcite (5)	3	7.0	1.025	Struvite (5), calcite (1)
4	8.5	1.014	Calcite (5), COD (2)	4	7.5	1.018	Struvite.(5), calcite(1), amorph. (5)
5	8.5	1.019	Calcite (5	5	7.5	1.016	Struvite (5), calcite (2)
6	8.5	1.019	Calcite (5)	6	8.0	1.017	Struvite (5), amorphous (5)
7	7.0	1.021	Calcite (1), COD (1)	7	7.5	1.017	Struvite (5), calcite (5), amorph.(5)

^a^ Calcite (calcium carbonate)—COD (calcium oxalate dihydrate)—Struvite (magnesium ammonium phosphate). ^b^ The abundance of crystals was assessed using a scoring system ranging from 1 to 5: score 1: 1–2 crystals, 2: 3–5 crystals, 3: 6–20 crystals, 4: 21–100 crystals; 5 > 100 crystals per microscopic field.

**Table 6:** Urinalysis of samples taken from fattening pigs on farms A and B and a control farm.

Parameter	Unit	Farm					
		A (n = 5)		B (n = 7)		Control (n = 15)	
		Mean	Median	Mean	Median	Mean	Median
Ca	mmol/g creatinine	6.34	6.80	0.48	0.44	1.80	1.68
P		0.83	0.87	4.32	4.33	1.19	0.41
Mg		9.19	9.23	1.27	1.41	3.19	2.61
Na		26.85	23.07	39.99	35.76	18.01	13.14
Cl		29.84	26.21	27.45	30.74	29.21	22.62
K		78.52	81.39	28.44	27.38	35.0	32.9
Citrate		2.29	2.61	0.11	0.12	0.18	0.16
Citrate	mmol/L	3.71	4.16	0.25	0.23	0.74	0.70
Creatinine	g/L	1.77	1.62	2.25	2.21	4.18	4.31

## Data Availability

Data are contained within the article.
